# Impacting Rare Diseases Healthcare by Case‐Based Telelearning: Description of the Dutch Pilot Metabolic Diseases ECHO

**DOI:** 10.1002/jmd2.70121

**Published:** 2026-10-08

**Authors:** Tirza Boschma, Désirée Y. van Haaften‐Visser, Maaike C. de Vries, Roos Dijkstra, Amaya Zinck, Esmeralda Oussoren, Terry G. J. Derks, Andrea B. Haijer‐Schreuder

**Affiliations:** ^1^ University Medical Center Groningen, Section of Metabolic Diseases University of Groningen, Beatrix Children's Hospital Groningen the Netherlands; ^2^ Department of Pediatrics, Center for Lysosomal and Metabolic Diseases, Erasmus MC Sophia Children's Hospital Rotterdam the Netherlands; ^3^ Section of Metabolic Diseases, Radboud University Medical Center Amalia's Children's Hospital, University of Nijmegen Nijmegen the Netherlands

**Keywords:** ECHO model, healthcare professionals, inherited metabolic diseases, knowledge dissemination, rare diseases, telelearning

## Abstract

Inherited metabolic diseases (IMDs) are individually (ultra)rare but collectively relatively common. The Extension for Community Healthcare Outcomes (ECHO) model has been used worldwide since 2003 to disseminate knowledge and best practices in chronic diseases. In 2024, the Dutch Society for Inborn Errors of Metabolism (ESN) launched a pilot Metabolic Diseases ECHO Program. We aimed to evaluate whether ECHO is a feasible model for IMD knowledge dissemination. We conducted a retrospective review of the design, implementation, and impact of the program. Since February 2025, two ECHO hubs have been established in two Dutch medical education and training regions (ETRs). Data have been prospectively collected through the iECHO platform. Program impact was assessed using Moore's Expanded Outcomes Framework, covering seven levels ranging from participation and satisfaction to competence development and population outcomes. The Metabolic Diseases ECHO Program has achieved broad geographic reach and strong engagement among healthcare professionals. To date, 15 sessions have been completed and 10 additional sessions are scheduled. A total of 208 professionals from diverse disciplines, including pediatrics, clinical genetics, clinical chemistry, and nursing, have participated, representing 10 of the 12 Dutch provinces. The program has stimulated the development of two regional treatment protocols and integrates all core academic tasks: patient care, education, and research. The ECHO model is a feasible and sustainable approach for disseminating expertise on IMDs. Our program demonstrates meaningful impact across the first four levels of Moore's framework. Long‐term continuation and expansion to other Dutch‐speaking regions are under consideration. Future work will focus on assessing the direct benefits for IMD patients.

## Key‐Points

- The Metabolic Diseases ECHO pilot program demonstrates that telelearning is a feasible, acceptable, and potentially transformative approach to address the educational and clinical challenges inherent to IMD care.
- Case‐based telelearning enabled broad dissemination of metabolic expertise beyond academic referral centers.
- More than 200 healthcare professionals from diverse disciplines participated during the first pilot year.
- Participants reported improved confidence in metabolic diagnostics and clinical decision‐making.
- The program initiated regional quality‐improvement activities, including the development of standardized metabolic care protocols.

## 1 Introduction

Inherited metabolic diseases (IMDs) comprise a heterogeneous group of more than 1900 rare genetic diseases caused by the defective function or absence of enzymes, transporters, or cofactors, leading to (toxic) accumulation or deficiency of specific metabolites [1]. Although most IMDs are individually ultra‐rare, their collective incidence was already estimated at 1:800 before the advent of exome sequencing [2]. Globally, IMDs represent the largest group of monogenic inherited diseases and account for at least 23 529 deaths annually among children under 5 years of age [3]. Their rarity, biochemical complexity, and broad clinical spectrum demand specialized clinical and diagnostic expertise, coordinated multidisciplinary management, and long‐term follow‐up.

However, delays in diagnosis, treatment, and access to specialized expertise are common due to the rarity and complexity of IMDs. IMDs are scarcely addressed in standard medical curricula and postgraduate training, resulting in limited awareness among healthcare professionals. Furthermore, in the Netherlands, as in many countries, expertise in IMDs is concentrated in a limited number of specialized academic medical centers [4]. Therefore, IMD expertise is frequently not available near the patient and their family, creating barriers to timely and equitable care [5].

Since 2003, the Extension for Community Healthcare Outcomes (ECHO) model has been used worldwide to disseminate specialist knowledge and best practices for numerous chronic and complex diseases [6, 7]. Project ECHO is a telelearning model designed to democratize specialist knowledge by connecting expert “hubs” (multidisciplinary teams at specialized centers) with community‐based “spokes” (clinicians who participate in the sessions) through regular virtual sessions. Using brief didactic presentations and case‐based discussions, the model fosters a community of practice in which all participants teach and learn from one another. ECHO has been successfully applied worldwide. To date, there are 8823 ECHO programs in 215 countries and areas for 8 935 000 ECHO session attendances (https://projectecho.unm.edu/; Accessed on 19‐02‐2026). Interestingly, a minority of these ECHO programs and ECHO hubs is active in the European Union. To the best of our knowledge, there are only a few ECHO hubs/programs dedicated to rare diseases, including rare bone diseases, intestinal failure, Prader‐Willi syndrome, Batten disease, and Ehlers‐Danlos syndromes (Table 1).

**TABLE 1:** ECHO programs in rare diseases.

Rare disease area	Country/region	Primary participants	Key reported outcomes	References
Rare bone diseases	United States	Endocrinologists, pediatricians	Improved protocol adherence; increased self‐reported competence	Tosi et al. [8]
Prader–Willi syndrome	United Kingdom	Pediatricians, dietitians	Enhanced multidisciplinary coordination; earlier specialist referral	Rujeedawa et al. [9]
Batten disease	United States	Neurologists, nurses	Improved recognition of early symptoms; more uniform care pathways	Scherr et al. [10]
Ehlers–Danlos syndromes	United States	General practitioners, rehabilitation physicians	Reduced professional isolation; high participant satisfaction	Schubart et al. [11]
Intestinal failure	United States	Dietitians, physicians, pharmacist and nurses	Improved knowledge; interprofessional knowledge sharing	Winkler et al. [12]

To address the educational and clinical gaps in IMD care, the Dutch Society for Inborn Errors of Metabolism (ESN) launched a two‐year pilot Metabolic Diseases ECHO program. Implemented in parallel across two medical education and training regions (ETRs; in Dutch: opleidings‐ en onderwijsregio's (OORs)) in the Netherlands (OOR Noordoost and OOR Zuidwest and Leiden), the program aimed to (1) strengthen collaborative learning through best practice and case‐based discussions and expert‐supported peer exchange and (2) establish a sustainable national model for sharing IMD expertise. We questioned if our ECHO program is feasible and sustainable for sharing expertise in IMDs in the Netherlands. This article describes the program's structure, participation, early evaluation outcomes, and emerging quality improvement initiatives.

## 2 Methods

### 2.1 Dutch Society for Inborn Errors of Metabolism

The ESN, founded in 1988, is a multidisciplinary professional society with approximately 180 members. Its membership includes metabolic pediatricians, metabolic internists, (pediatric) neurologists, clinical geneticists, diagnostic laboratory specialists, dietitians, researchers, and patient representatives. In addition to Dutch institutions, members are also affiliated with centers in the Dutch‐speaking regions of Belgium. Most ESN members work in academic medical centers and contribute to clinical care, research, and education in IMDs.

### 2.2 Organization of Healthcare for Inherited Metabolic Diseases in The Netherlands

The Netherlands is divided into seven ETRs, each coordinated by one of the seven University Medical Centers (UMCs). Within each ETR, UMCs collaborate with regional hospitals and training institutions to provide internships for medical students, residency programs, and postgraduate education for medical specialists, general practitioners, clinical chemists, clinical physicists, and hospital pharmacists.

Referral patterns for suspected IMDs largely align with these geographically organized ETRs, including referrals following abnormal population newborn screening results. Six UMCs host specialized metabolic departments: Amsterdam UMC, Erasmus MC, Maastricht UMC+, Radboud UMC, UMC Groningen, and UMC Utrecht. Erasmus MC coordinates both the ETRs Southwest Netherlands and Leiden.

Since 2015, the Netherlands has recognized approximately 350 Centers of Expertise (CEs) for rare diseases, several of which focus on specific (groups of) IMDs. Specific examples of IMD CEs for (groups of) IMDs include peroxisomal diseases (ORPHA:68373) in Amsterdam UMC, lysosomal storage disorders (ORPHA:309337) in Erasmus MC, disorders of galactose metabolism (ORPHA:308467) in Maastricht UMC+, mitochondrial diseases (ORPHA:68380) in Radboud UMC, glycogen storage diseases (ORPHA:79201) in UMC Groningen, and (long‐chain) fatty acid oxidation disorders (ORPHA:26793) in UMC Utrecht.

### 2.3 Design and Launch of the Pilot Metabolic Diseases ECHO Program

Coordinated from and on behalf of the ESN (specifically the education committee, in collaboration with the board and the sponsorship committee), the initiative was taken by the UMCG (TGJD and ABHS; acting as program directors and local facilitators) and Erasmus MC (DHV and EO; acting as program directors and local facilitators) to design the Metabolic Diseases ECHO Program. UMCG and Erasmus MC are coordinating ETR Northeast Netherlands (abbreviated as NE) and Southwest Netherlands and Leiden (SWL), respectively. Program coordination was performed by Congreslink (https://www.congreslink.nl/) and data collection was supported by trained medical students from the UMCG (TB, RD, and AZ).

Table 2 presents the milestones of the design and implementation of the Metabolic Diseases ECHO Program.

**TABLE 2:** Milestones of the metabolic diseases ECHO program.

Date	Milestone
23 March 2023	Discussion during ESN members' meeting of the topic “Metabolic Diseases ECHO Program” and conditions for sponsorship by private parties
02 June 2023	Online meeting between representatives of the ESN board, ESN Training Committee, ESN Sponsorship Committee and ESN sponsors
27 March 2024	Signed “Project ECHO Intellectual Property Terms of Use Agreement”
March–June 2024	Signed contracts between ESN and 4 ESN‐sponsors
09 July 2024	Stakeholder kickoff meeting
24 February 2025	First session by UMCG in ETR‐NO
27 May 2025	First session by ErasmusMC in ETR SWL
01 July 2025	Stakeholder evaluation meeting (hybrid)

The local facilitators of both ETR NE and ETR SWL organized online metabolic continuing education (CE) in the form of this Metabolic Diseases ECHO as a pilot project in 2025 and 2026. Our ECHO project provided CE credits at no cost to participating physicians.

The target audience was pediatricians, internists, (pediatric) neurologists, clinical geneticists, clinical chemists, and residents of these specialties, but other interested healthcare providers are also welcome to participate.

### 2.4 Program Description

Before we were able to launch our ECHO project, an extensive preparatory phase was undertaken. As shown in Table 2, multiple stakeholder meetings were held, and the hub coordinators received comprehensive training to qualify them to lead an ECHO session.

The pilot program consisted of (data reported for the period February–December 2025):

- 15 monthly sessions
- Session duration: 2 h.
- Format: 1 evidence‐based didactic lecture + 1 de‐identified case discussion.
- Disciplines represented: Pediatricians, Internists, Clinical Geneticists, Dietitians, Nurses, Clinical chemists, Physicians for people with intellectual disabilities.
- Across the 15 sessions, participants presented 15 clinical cases and attended 15 didactic lectures, demonstrating consistent engagement with both core components of the ECHO model.
- The sessions were accredited with 2 continuing medical education (CME) credits.
- More details are shown in Table 3.
- Participants were recruited via save‐the‐dates combined with additional information about our online Metabolic Diseases ECHO program, sent to all secretaries of pediatric, internal medicine, neurology, clinical chemistry, and clinical genetics departments. Secretaries were asked to distribute our invitations among the clinical doctors and department members. Also known email lists (e.g., from our ETR referee evenings) were used. Furthermore, our save‐the‐dates were shared on social media and the ESN website.

**TABLE 3:** Data of the metabolic diseases ECHO program.

	Education and training regions		
	UMCG	Erasmus MC	Totals
First session (dd/mm/yyyy)	24/02/2025	27/05/2025	
Sessions conducted (*n*)^a^	9	6	15
Sessions scheduled (*n*)^a^	5	5	10
Registered participants (*n*)	147	119	266
Tota l attendances (*n*)	138	70	208
Total participants learning sites (*n*)^b^	36	27	63

^a^ Sessions scheduled' includes all planned sessions at the time of writing; “sessions conducted” includes only the sessions that had already taken place and were included in the evaluation.

^b^ Participants' learning sites' refers to the hospitals or organizations where participants work.

### 2.5 Evaluation of the Pilot Metabolic Diseases ECHO Program

Evaluation of the pilot program followed the framework of Moore et al. [13], which assesses outcomes across seven levels of CE effectiveness:

- Participation: The number of participants was evaluated using data from the iECHO platform, in which participants are required to register prior to each ECHO session.
- Satisfaction: A structured evaluation was conducted using standardized online questionnaires administered at the end of the first four sessions. The balance between presentation and interaction was evaluated. Also, a Grade (1–10) was asked about the likelihood of suggesting the ECHO program to a colleague.
- Learning: To assess short‐term learning outcomes, pre‐ and post‐session knowledge assessments were administered using brief digital questionnaires consisting of 5 multiple‐choice questions directly aligned with the learning objectives of five sessions (Sessions 1, 2, 3, 4, and 8). These assessments were completed immediately before and after the session, respectively. The pre‐ and post‐test items were developed by the SME, based on the rationale that the SME possesses both the medical expertise required to construct high‐quality assessment questions and a detailed understanding of the content covered in the presentation. Participation was voluntary.
- Competence: A question about how competent attendees felt about the subject discussed during the ECHO session was evaluated based on a mark (range 1–10) during the first four sessions. All session attendees were invited to complete the post‐session questionnaire; participation in the evaluation was voluntary.
- Performance: this level is not formally evaluated during the first pilot year
- End‐beneficiary outcomes (impact on patient care): of this level early observations are shared
- Population outcomes: of this level early observations are shared.

### 2.6 Analysis of Pre‐ and Post‐Test Results

The analysis of the medical knowledge test results was conducted using the Statistical Package for the Social Sciences (SPSS). Prior to the analysis, a normality test was performed to determine whether the data were normally distributed. If the data were normally distributed (*p* > 0.05), a paired *t*‐test was subsequently applied. In cases where the data were not normally distributed, a non‐parametric Wilcoxon signed‐rank test was used instead.

These tests were employed to determine the level of statistical significance of changes in medical knowledge before and after the session. A *p* value of less than 0.05 was considered statistically significant. In addition, the percentage change in scores between the pre‐test and post‐test was calculated.

## 3 Results

In the following section, we will evaluate the outcomes following Moore's Expanded Outcomes Framework.

### 3.1 Participation

Participation was strong throughout the program. Recruitment was conducted through a formal dissemination process as described in the methods. In total 208 healthcare professionals are registered in the iECHO program. Attendance remained stable, with a mean of 15 (range 7–24) participants per session, and most participants attending the full two‐hour sessions (Table 4). The multidisciplinary composition of the group reflects the complexity of IMDs and the need for coordinated care across specialties.

**TABLE 4:** Agenda of the metabolic diseases ECHO program.

Session	Date	ETR	SME topic	Attendance	Learning		
					Pre	Post	Both
1	24/02/2025	NO	Emergency protocols	14	11	11	11
2	31/03/2025	NO	Hypoglycemia	19	8	7	5
3	14/04/2025	NO	Genetic testing	14	11	10	9
4	26/05/2025	NO	Newborn screening	13	6	5	4
5	27/05/2025	SWL	Hypoglycemia	7			
6	23/06/2025	NO	Metabolic “reg flags”	11			
7	24/06/2025	SWL	Acute metabolic problems	17			
8	22/09/2025	NO	Developmental delay	24	15	11	10
9	23/09/2025	SWL	Genetic testing	15			
10	14/10/2025	SWL	Liver problems	9			
11	27/10/2025	NO	General clinical chemistry applied to metabolic diseases	15			
12	18/11/2025	SWL	Developmental delay	21			
13	24/11/2025	NO	Hepatomegaly	18			
14	15/12/2025	NO	Core metabolic diagnostics	22			
15	16/12/2025	SWL	Lysosomal storage diseases	10			

The program has successfully engaged multiple regional hospitals within the designated training regions, as illustrated in Figure 1. The figure presents a map of the Netherlands on which circles indicate the participating (general) centers.

**FIGURE 1:**
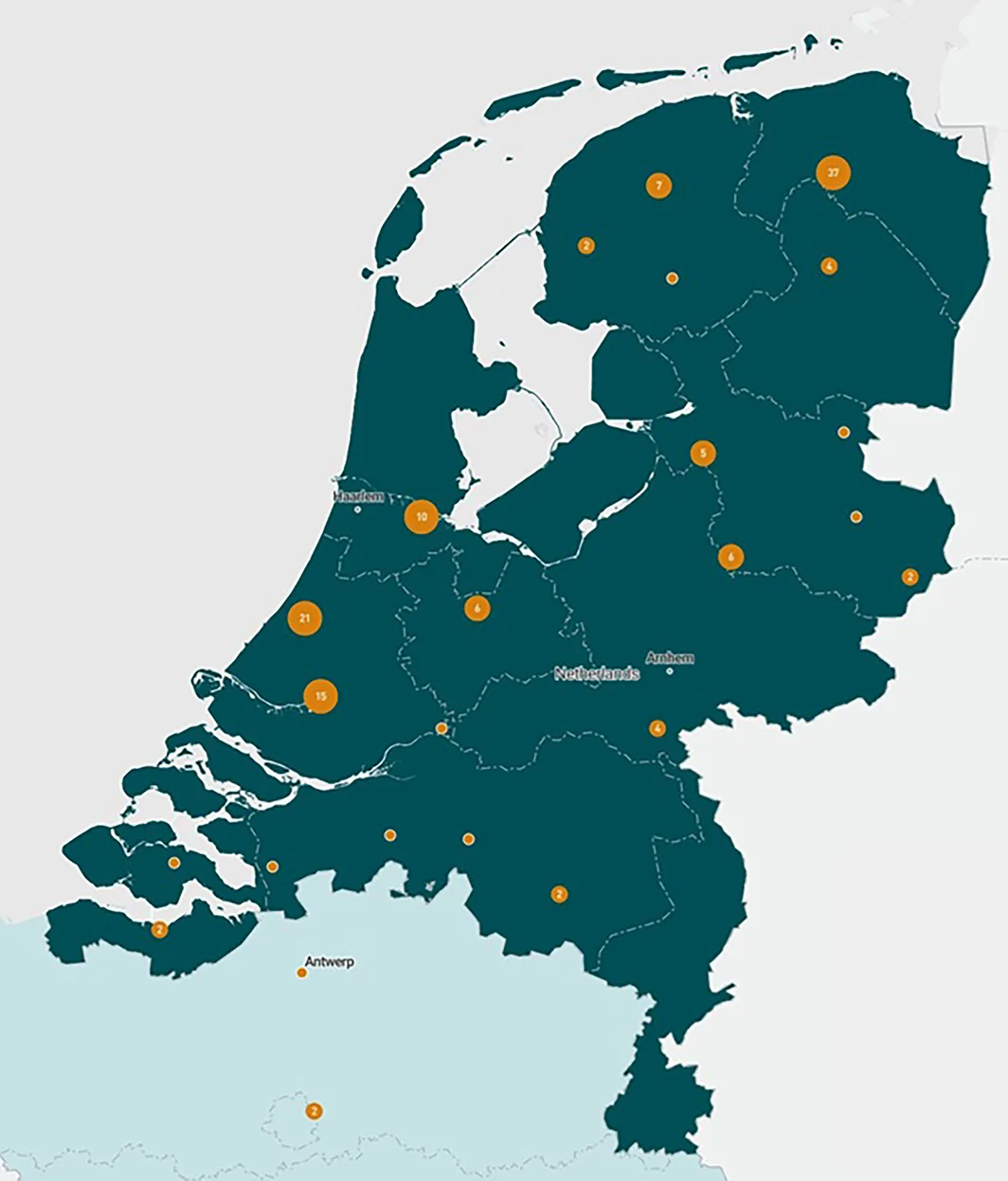
Participants' locations after the first pilot year of the Metabolic Diseases ECHO.

### 3.2 Satisfaction

Participants were invited to complete a questionnaire (File S1) immediately following each session (applicable for the first four sessions). Response rates were high, > 90%. An average of 85.1% of participants reported that the balance between case‐based discussion and the in‐depth subject matter expert presentation was appropriate. When asked which part of the ECHO session they found most useful, the responses were grouped into four overarching themes: the didactic presentation, the case presentation, interaction, and practical tips. In Session 1, participants most frequently identified the didactic presentation as the most useful component (66.6%). In Session 2, both the didactic presentation (42.9%) and the practical tips (42.9%) were rated as equally useful. In Session 3, participants again considered the didactic presentation to be the most useful element (53.9%), followed by the case presentation (30.8%). In Session 4, the case presentation was regarded as the most useful component by the majority of participants (80.0%).

When asked whether they would recommend an ECHO session to a colleague, respondents provided an average score of 8 on a 1–10 scale (range 6–9). The mean score for Session 1 was 7.1 (median = 7, SD = 0.9). For Session 2, the mean score increased to 8.2 (median = 8, SD = 0.7). Session 3 showed a comparable result, with a mean score of 8.0 (median = 8, SD = 0.9). In the final session, the mean score decreased to 7.6 (median = 8, SD = 0.5).

### 3.3 Learning

Using pre‐ and post‐session knowledge assessments, we sought to quantify the immediate learning gains associated with each session. All attendees were invited to complete a pre‐ and post‐session assessment during five of our ECHO sessions. Response rates are shown in Table 5. Participation was voluntary. In total 51 pre‐tests, 44 post‐test and 39 as well pre‐ and post‐test results were collected (Table 4). The results of the medical knowledge assessments showed a modest increase in knowledge levels. In Session 1, the average score improved by 3.0%. In Session 2, a slight decrease of 1.3% was observed. In contrast, Session 3 demonstrated an increase of 7.7%. The largest improvement was recorded in Session 4, where the average score increased by 57.7%. Finally, in Session 8, the average score increased by 45%.

**TABLE 5:** Response rate of the pre‐ and post‐assessment per ECHO session.

Respons	Pre‐test (%)	Post‐test (%)	Both (%)
Session 1	80.0	80.0	73.3
Session 2	44.4	50.0	27.8
Session 3	78.6	85.7	64.3
Session 4	66.7	80.0	57.1
Session 8	78.9	77.8	52.6

Participants who achieved the maximum possible score on both the pre‐test and post‐test (ceiling effect) were excluded from the analysis. After this correction, the results for sessions one and two remained unchanged. However, the average increase in session three rose to 13.0%. This correction was not applicable to sessions four and eight, as no participants achieved the maximum score at baseline.

The analysis of the medical knowledge tests (*N* = 39) showed no statistically significant difference between the pre‐test and post‐test measurements (*p* = 0.18), indicating that the ECHO sessions did not result in a statistically significant change in medical knowledge.

Additionally, it is informative to present the topics that were offered to participants for educational purposes. These are displayed in Table 4. The topics discussed vary by ETR, as they are selected based on current clinical cases, the availability of subject matter experts, and the specific requests of participants.

### 3.4 Competence

After each session, participants were asked to rate their own competence regarding the topic discussed during each session with a mark (range 1–10) (only applicable for the first four sessions). Participants reported enhanced confidence in the metabolic cases discussed during the sessions. Also, they felt more confident in areas such as interpretation of biochemical profiles, metabolic or genetic diagnostic tests, and recognition and treatment of the metabolic diseases discussed during the subject matter expert sessions. On average, participants rated their competence at 7.6 following Session 1, 6.3 following Session 2, and 6.8 following Session 3. In Session 4, the average self‐rated competence score was 7.0. Several respondents noted that the case‐based discussions helped them translate theoretical knowledge into practical decision‐making, and that the interactive format reduced the threshold to ask questions, including for future professional peer consultation.

### 3.5 Performance

A formal evaluation of practice change is planned for the next program cycle. Early informal observations point to an increased initiation of basic metabolic diagnostics by general hospital clinicians and have increased the frequency of multidisciplinary consultations. Many emphasized that the ECHO model facilitated cross‐regional collaboration. These findings will be explored in greater depth through structured follow‐up assessments.

### 3.6 End Beneficiary‐ and Health Outcomes

The Metabolic Diseases ECHO program has served as a catalyst for several emerging quality improvement initiatives across participating institutions. Although these projects are in early stages, initial observations indicate that the program has stimulated renewed attention to workflow optimization, interdisciplinary communication, and the integration of metabolic expertise into routine clinical practice.

At least three participating sites have begun developing or refining standardized protocols for metabolic screening, for example local protocols for the laboratory work‐up during hypoglycemia. Others have initiated efforts to have a closer look at the emergency protocol during a metabolic crisis. These changes appear to be facilitated by the shared learning environment of the ECHO sessions, where participants exchange practical strategies for implementation and troubleshooting.

## 4 Discussion

This pilot Metabolic Diseases ECHO program demonstrates that it can be feasible and sustainable to use the ECHO model for disseminating expertise in diagnosing and managing IMDs, a domain in which expertise is scarce and unevenly distributed. Participation was strong and sustained throughout the first year, consistent with findings from ECHO programs and other telehealth‐based models in other rare disease areas, where high engagement has similarly been reported [8, 9, 10, 11, 12].

High levels of participant satisfaction indicate that the program's structure, combining case‐based discussions with didactic teaching, was well aligned with participants' needs. This mirrors outcomes from ECHO initiatives in rare bone diseases and Prader–Willi syndrome, where participants valued the opportunity to discuss complex cases with experts and peers [8, 9]. International studies have shown that ECHO programs strengthen multidisciplinary connectedness and create a shared learning culture among clinicians caring for rare diseases [14]. Our findings reflect this pattern, with participants frequently highlighting the value of cross‐regional peer exchange.

Although pre‐ and post‐session knowledge assessments did not show differences, this pattern is consistent with the broader ECHO literature. Programs in rare endocrine and rare bone conditions often report modest short‐term knowledge gains but more substantial improvements in clinical confidence and decision‐making [8, 9]. In our evaluation, the absence of pre‐post differences should be interpreted with caution, as the sample size was relatively small and participants may already have possessed a solid baseline level of IMD‐related knowledge. Another explanation might be that participation in the program was voluntary, and it is possible that clinicians with a particular interest in IMDs were more likely to participate. This combination likely reduced the sensitivity of the assessments to detect change. Nonetheless, participants reported increased confidence in interpreting biochemical profiles, selecting diagnostic tests, and managing the IMDs discussed.

Early indications of best‐practice improvement/practice change were also observed. Participants described adopting more standardized approaches to metabolic screening and increasing the frequency of multidisciplinary consultations. Although not an ECHO program, comparable shifts have been documented in the telehealth‐based model for Batten disease, where improved protocol adherence and earlier specialist referral were reported [10]. The emergence of quality improvement initiatives in our program, such as refining hypoglycemia work‐up protocols and reviewing emergency protocols for metabolic crises, emphasized these findings.

Several limitations must be acknowledged. The evaluation period was relatively short, and many outcomes, particularly those related to performance, patient health, and population health, require longer follow‐up, a challenge also noted in other rare disease ECHO projects [9, 11]. The absence of significant pre‐post knowledge gains may reflect limitations in assessment tools or ceiling effects among participants with prior IMD exposure, a phenomenon described in other ECHO programs for rare genetic disorders [9]. Participation was voluntary, which may have introduced selection bias, and some professional groups remained underrepresented, as also reported in international ECHO literature [8, 9]. In our cohort, dietitians, psychologists, and laboratory specialists were underrepresented compared with physicians and nurse specialists. This may be related to workload constraints, limited protected time for educational activities, or lower initial awareness of the program. The lower initial awareness could be due to our recruitment strategy. We tried to reach a broad public and by sending the invitation ourselves we hoped to create a low threshold for participants. However, using our own network could lead to self‐selection bias. Potential approaches for future sessions could include targeted outreach and scheduling sessions at times that better accommodate these professional groups.

To ensure long‐term success, further strengthening of the evaluation framework will be essential, through longitudinal tracking of practice changes, structured follow‐up surveys, and integration of clinical outcome indicators. Rare‐disease ECHO programs increasingly incorporate mixed‐methods evaluation and patient‐reported outcomes [14], and adopting similar strategies may enhance the validity of future assessments. Expansion of the Metabolic Diseases ECHO program to additional regions in Dutch‐speaking areas and broader inclusion of professional disciplines are currently being explored to increase the program's reach and sustainability. Harmonizing care pathways across centers, supported by shared protocols developed within the ECHO community, represents a promising avenue for improving equity and consistency in IMD care nationwide. These ambitions are in line with the main objective of the European Reference Networks (ERNs), which have been established since 2017 to improve care for patients with rare or complex conditions by pooling expertise across national borders. To date few ECHO hubs exist within the EU rare diseases community, and we hypothesize whether the model is scalable to contribute to the ERN ambitions. The ECHO model is intentionally designed to be scalable and adaptable across diverse health‐care contexts. The core components: hub‐and‐spoke structure, case‐based learning, and multipoint videoconferencing, are technology‐agnostic and have already been successfully implemented in multiple countries and clinical domains. Based on this global track record, it is reasonable to conclude that the model is feasible and applicable for IMD programs in other countries, provided that local stakeholders ensure minimal digital infrastructure, identify a coordinating hub, and tailor the curriculum to local needs. In parallel, future work will address the direct clinical benefit for IMD patients themselves.

Future quality improvement efforts will focus on further harmonizing care pathways across regions and strengthening the consistency of documentation and metabolic outcome data. In contrast to the Clinical Patient Management System (CPMS), which facilitates case‐based consultation at the European level, our ECHO Program enables structured, recurring knowledge exchange within the Dutch UMC network, allowing multidisciplinary teams to discuss complex IMD cases with the relevant national expertise center acting as a subject matter expert. Another possible use of the ECHO Program is the possibility to discuss complex metabolic patients among the UMCs, with the subject matter expert being the medical specialist from the expertise center relevant to the case. By embedding these discussions into regular online learning sessions within our ETRs, the Metabolic Diseases ECHO Program is expected to remain a central driver of national alignment and continuous professional development.

In conclusion, the Metabolic Diseases ECHO pilot program demonstrates that telelearning can be a feasible, acceptable, and potentially transformative approach to address the educational and clinical challenges inherent to IMD care. Our pilot ECHO program showed several positive examples of practice change. Our findings align closely with international experiences in rare disease ECHO programs, which collectively show that ECHO can strengthen clinician confidence, foster multidisciplinary collaboration, and stimulate quality improvement [11, 14]. Continued refinement, expansion, and evaluation of the effects will be crucial to realizing its full potential and ensuring lasting improvements in the quality of metabolic care in the Netherlands.

## Author Contributions

Terry G.J. Derks and Andrea B. Haijer‐Schreuder developed the initial idea for an ECHO program in metabolic diseases. Terry G.J. Derks, Andrea B. Haijer‐Schreuder, Désirée Y. van Haaften‐Visser, and Esmeralda Oussoren shaped and implemented the ECHO program in The Netherlands. All authors contributed to the online training sessions, either through content development, chairing sessions, or providing ICT support. Tirza Boschma, Andrea B. Haijer‐Schreuder, and Terry G.J. Derks wrote the first draft. Tirza Boschma, Andrea B. Haijer‐Schreuder, Amaya Zinck, and Roos Dijkstra analyzed the data. All authors provided critical feedback to improve the manuscript. All authors approved the final manuscript as submitted and agreed to be accountable for all aspects of the work. All authors confirm the absence of previous similar or simultaneous publications.

## Funding

The ECHO project itself was sponsored by the Dutch Society for Inborn Errors of Metabolism (ESN), as stated in the manuscript. The sponsors were not involved in the content of the ECHO sessions nor in the content of this manuscript.

## Ethics Statement

The authors have nothing to report.

## Conflicts of Interest

The authors declare no conflicts of interest.

## Supporting information


**File S1:** ECHO feedback form ETR NO.

## Data Availability

The data that supports the findings of this study are available from the corresponding author upon reasonable request.
