# Supplementary material for: Impacting Rare Diseases Healthcare by Case‐Based Telelearning: Description of the Dutch Pilot Metabolic Diseases ECHO
Source: JIMD Rep. 2026 Oct 8;67(6):e70121. doi: 10.1002/jmd2.70121 (PMC13647106; doi:10.1002/jmd2.70121)
Supplement: Supplementary file 1 [file JMD2-67-e70121-s001.pdf]

ECHO ETR NO Feedback

Thank you for participating in this ECHO session.

We appreciate your feedback through this short 5-minute survey.

1. Consent to use responses for research

- I agree that Project ECHO may use my responses in its research project.
- I do not agree, but I would still like to provide feedback.

2. What is your opinion on the balance between presentation and interactivity in this session?

- Too much presentation and too little interactive learning
- Good balance between presentation and interactive learning
- Too little presentation and too much interactive learning

3. After this ECHO® session, I feel more competent regarding the topic discussed.*

- Yes
- No

4. Based on the knowledge gained in this session, do you expect to better recognize and respond to the session topic in the future?

- Very likely
- Likely
- Possible
- Unlikely
- Very unlikely

5. How likely are you to recommend this session to a colleague?

- Not at all
- Unlikely
- Likely
- Definitely

6. Which part of this session was most useful for your learning?

7. How could this session be improved to provide a more effective learning experience?

8. What topics would you like to learn more about in the future?
